# International study on the prevalence of malnutrition in centralized care for colorectal cancer patients

**DOI:** 10.1515/iss-2023-0015

**Published:** 2023-06-13

**Authors:** Carl Meissner, Svenja Tiegges, Martin Broehl, Ronny Otto, Karsten Ridwelski

**Affiliations:** Klinikum Magdeburg gGmbH, Klinik für Allgemein- und Viszeralchirurgie, Magdeburg, Germany; An-Institut für Qualitätssicherung in der operativen Medizin gGmbH an der Otto-von-Guericke Universität Magdeburg, Magdeburg, Germany; WissWerk, PubliCare GmbH, Cologne, Germany; Medizinischen Versorgungszentrum „Im Altstadtquartier“ GmbH, Haus- und Facharztzentrum, Ambulantes Operationszentrum, Magdeburg, Germany

**Keywords:** colorectal cancer center, colorectal carcinoma, length of stay, malnutrition, NRS screening, nutrition management, SGA score

## Abstract

**Objectives:**

Patients with different diseases may show signs of malnutrition both before and during the hospital stay. The presence of malnutrition may impact the recovery and length of stay and consequently the costs. Early identification of malnutrition is thus a critical factor. The objective of this multicenter study is to determine the prevalence of malnutrition in colorectal cancer centers. Another objective is to investigate possible consequences, such as complications or length of stay. In addition, the study aims to demonstrate the relevance of nutrition management in colorectal cancer centers. At the same time, relevant requirements clearly demanded by the Certification Commission for Certified Colorectal Cancer Centers are met through implementation of the study.

**Methods:**

Between 2019 and 2021, patients in colorectal cancer centers were examined in the preoperative phase. In addition to questions about patients’ state of health and nutrition, the validated screening forms—Subjective Global Assessment (SGA) and Nutritional Risk Screening Tool 2002 (NRS 2002)—were used to assess malnutrition. The data were processed by univariate analysis.

**Results:**

In total, data records of 3,102 patients were evaluated. The mean age of the participants was 68.5 ± 11.9 years, and their average body mass index (BMI) was 26.8 ± 5.3 kg/m^2^. The SGA questionnaire indicated that 23 % of the participants suffered from malnutrition and 38 % were at risk of malnutrition (NRS≥3). Malnutrition was found more frequently in patients with colorectal carcinomas than in patients with rectal carcinomas (53.1 vs. 32.1 %). The length of stay in hospital and the rate of complications were significantly higher when malnutrition was identified.

**Conclusions:**

Approximately one in three to four patients with a colorectal carcinoma has an increased risk of malnutrition. The two screening methods calculated a different prevalence (23 and 38 %). Any malnutrition that is present demonstrably has a significant impact both on the rate of complications and the length of stay and may therefore have a decisive influence on the costs. The results of this multicenter study underscore the need for systematic screening for malnutrition and at the same time should increase clinics awareness of the importance of establishing a nutrition management policy.

## Introduction

Approximately one in eight cases of cancer in Germany involves the colon or rectum. In 2018, more than 60,000 people had this disease. According to the analysis of key indicators in the 2022 Annual Report of the Certified Colorectal Cancer Centers, 26,998 primary cases had been treated by late-2021 in all of the 301 certified colorectal cancer centers reviewed [1]. Special structural features for the care of patients are described in the survey form for certified colorectal cancer centers.

This survey form calls for structural and procedural features that go beyond the requirements in the Colorectal Carcinoma S3 guideline, also regarding preoperative identification of malnutrition. According to the requirement, the metabolic risk should be assessed using the Nutritional Risk Screening (NRS) tool at the latest on admission to hospital, for all cancer patients if possible, and any nutritional advice derived from this should be appropriately verified [2].

The subject of malnutrition is not even consistently addressed in all clinics or colorectal cancer centers. In this regard, the presence of malnutrition plays a relevant role, especially for oncological patients [3].

In the past, various studies have already shown that patients with different underlying diseases are malnourished both on admission and throughout their stay [4]. In their paper from 2014, Hébuterne et al. describe the prevalence of malnutrition in various tumor entities. In their random sample, the prevalence of malnutrition in colorectal carcinoma was 39 % [5]. Furthermore, any malnutrition may likewise have an impact on the length of stay in hospital and thus also plays a substantial role with respect to economic aspects.

Possible additional costs resulting from malnutrition were analyzed as early as 2007 [6]. The hospital, nursing, and outpatient sectors were considered in the calculation of these additional costs.

The costs result from—among other factors—the longer length of stay as well as additional nursing expenses. The changing age structure means that the number of patients requiring nursing care is increasing. The number of potentially malnourished persons increases in turn with the changing age structure. As an example for the outpatient sector, increased physician services and clinical nutrition products were included as costs. According to the CEPTON paper, the annual additional costs associated with these services and products were estimated to be €9 billion [6].

Malnutrition is a frequently occurring phenomenon in patients with chronic or consumptive diseases. Malnutrition is also regarded as an independent risk factor for the clinical outcome. Oncology patients experience altered physiological functions associated with weight loss, fatigue, or even psychological problems.

Nutrition therapy tailored to the patient demonstrably reduces the risk of mortality and improves the outcome for oncology patients [7].

Above and beyond the certification requirements of the German Cancer Society, the German Society for Nutritional Medicine also recommends the use of the Nutritional Risk Score [8]. This score considers various risk factors for malnutrition and is easily applied in routine clinical practice [9]. Likewise, the Subjective Global Assessment (SGA questionnaire) is suggested for estimating the nutritional status. The SGA is a reproducible method for estimating the nutritional status in both outpatients and inpatients [10].

The concept of prehabilitation in particular is another important factor in this case. Prehabilitation is defined as the process of improving the individual performance and functional capability prior to a planned surgical procedure with the aim of improving the tolerance of the patient to upcoming physical stresses [11].

These recommendations are found in the guidelines of the German Society for Nutritional Medicine (DGEM) and the European Society for Clinical Nutrition and Metabolism (ESPEN) for clinical nutrition in surgery. Numerous programs for enhanced recovery after surgery (ERAS) also contain relevant advice.

According to the S3 Guideline of the German Society for Nutritional Medicine (DGEM), it follows in summary that perioperative supplementary artificial nutrition is indicated even in patients without obvious malnutrition if the patient will be incapable of adequate oral intake of calories for a lengthy postoperative period [12], [13], [14].

## Materials and methods

### Patient population

The study was conducted between 2019 and 2021. In total, data records of 3,102 patients were evaluated. The patients were treated in colorectal cancer centers in Germany, Austria, and Switzerland. Only patients with colorectal carcinoma were included in the study.

### Screening for malnutrition and data collection

Two validated screening methods were used to assess a possible malnutrition risk.

Nutritional Risk Screening (NRS) considers various risk factors for malnutrition, and the respective risk of malnutrition is derived from the calculated scores [9]. It is recommended for the inpatient sector but is used internationally in various settings.

A further tool used during the study was the Subjective Global Assessment (SGA) tool [10]. From the medical history, various factors, including weight change, food intake, or gastrointestinal symptoms, are used by the investigator to estimate the nutritional status of the patient.

The two methods have different characteristics. The NRS score is subdivided into a preliminary and main screening. The risk of malnutrition is determined by a total score. The SGA score is determined both through questions about the current nutritional status and from physical examinations. The end result is estimated by the investigator on the basis of those components.

The results obtained from the screening methods are compared with the possible procedures of nutrition therapy and analyzed scientifically with further data from the patient documentation (age, sex, height, weight, residence type, diagnoses, habits, therapeutic procedures, and complications).

Data were collected prior to surgery and postoperatively. All data were transmitted to An-Institut and transferred to a database.

### Statistical analyses

The results of individual variables are plotted as absolute and relative frequencies and the comparison of two categorical variables is presented as a cross table. Pearson’s *χ* ^2^ test was applied to test the independence between variables, and the associated effect size was determined by Cramer’s V. The Mann–Whitney U test was used to test categorical variables with 2 levels in relation to metric variables. The rank biserial correlation (r) was used to calculate the effect size. The Kruskal–Wallis test was used for categorical variables with more than 2 features, and the effect size was measured as epsilon squared (ε^2^). A p-value of <0.05 was assumed to show statistical significance. Calculations and graphs were prepared with statistical software from the R Foundation [15]. The graphs were plotted using the ggstatsplot r-package [16]. Further graphs were plotted with Microsoft Excel (2016).

## Results

### Study population

The study participants included 1,273 women (41 %) and 1822 men (59 %). The patients had an average age of 68.5 ± 11.9 years (range, 25–97 years). In this study population, women were 1.8 years older on average (67.7 vs. 69.5 years). The average height was 171.6 ± 9.5 cm and the weight was 79.3 ± 17.5 kg. Men were approximately 13 cm taller than women and on average weighed 15.5 kg more. The mean body mass index (BMI) was 26.8 ± 5.3 kg/m^2^. According to the World Health Organization, this corresponds to overweight [17].

According to the classification, 50 patients (1.61 %) are underweight and 913 patients (29.43 %) have normal weight. Among the patients included in this study, 2053 (66.18 %) are in the overweight category [17].

In total, 127 clinics participated in the study, including 110 centers that were certified colorectal cancer centers at the time of the study. In addition to the 125 German clinics, one center each from Switzerland and Austria also participated in the study. These were also certified colorectal cancer centers that are likewise subject to the German certification process.

Over 85 % of the patients were diagnosed with colorectal carcinoma (53.1 %) or rectal carcinoma (32.2 %). The UICC classifications were distributed mainly between 1 and 3 (25 , 29, 25 %).

For most patients (74 %), no nutrition therapies were administered preoperatively. Approximately 13 % received liquid food and a further 5 % received enteral nutrition. Parenteral nutrition was administered in 1 % of the study patients.

## Screening methods

The nutritional status was determined using two validated screening methods.

**Figure 1:**
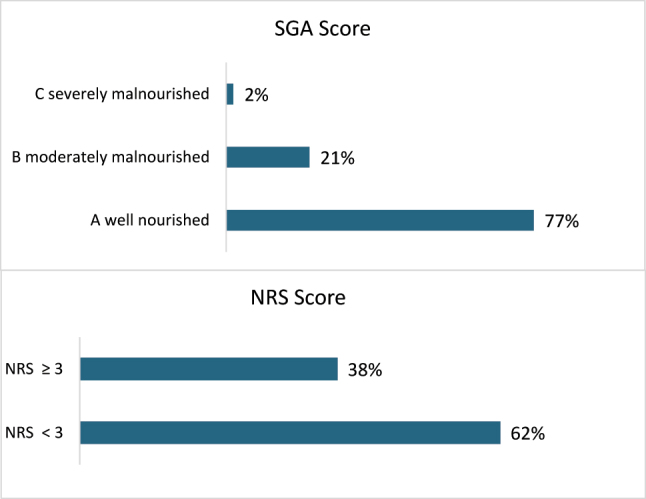
Distribution of the SGA screenings (n=3,025) and the NRS screenings (n=2,968).

According to the SGA score, 23 % (695 patients) showed signs of malnutrition. Based on the NRS scores, 38 % (1,122 patients) were at risk of developing malnutrition (Figure 1).

### Comparability of the two screening methods

The two screening methods identified different frequencies of malnutrition.

**Figure 2:**
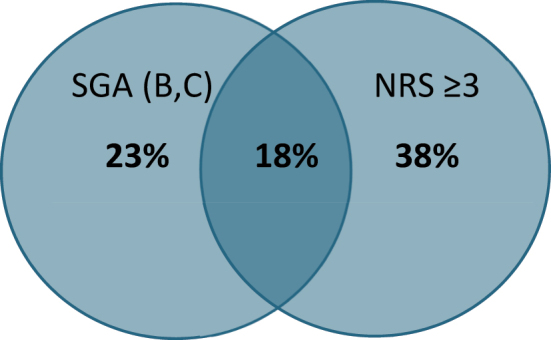
Venn diagram showing the results from both screening methods.

In 18 % (539 patients), malnutrition was detected by both screening methods (Figure 2). This means that both screening methods yielded a similar result for 539 patients. In this study population, malnutrition was identified more frequently by the NRS tool.

### Length of stay

The mean length of stay of all patients was 13.8 ± 9.9 days, with a minimum of 1 day and a maximum of 141 days.

**Figure 3:**
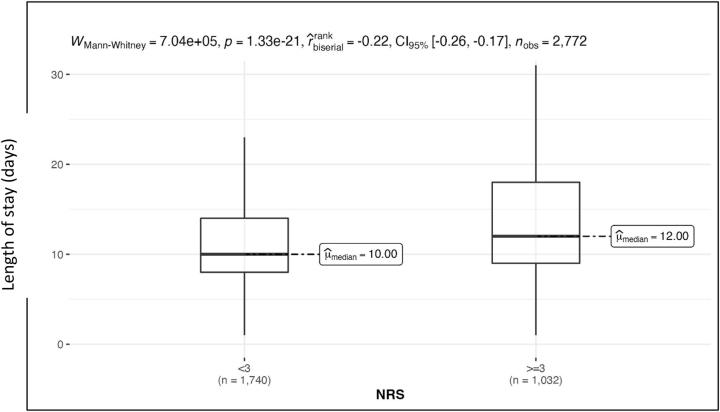
Length of stay as a function of the nutritional status determined according to NRS. p<0.001.

For patients who according to the NRS tool were not at risk of malnutrition, the average length of stay in the clinic was 12.6 ± 8.8 (median=10.0) days. For patients with an NRS score of ≥3, the average length of stay was 15.7 ± 13.2 (median=12.0) days, which was significantly longer (Figure 3).

**Figure 4:**
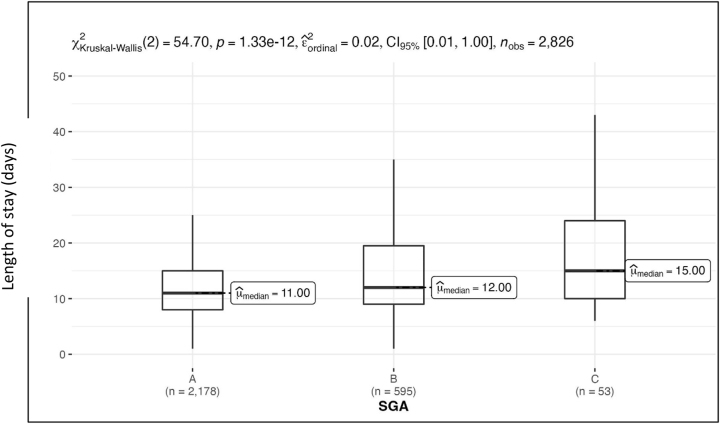
Length of stay as a function of the determined SGA score, p<0.001.

Figure 4 shows that the length of stay in the clinic depends on the nutritional status of the patients. Patients with an SGA score of A had an average length of stay of 13 ± 8.6 (median=11.0) days. The average length of stay in the clinic for moderately malnourished patients was 16.3 ± 12.9 (median=12.0) days. The longest length of stay in the hospital was found for the patient group with an SGA score of C. This corresponds to those patients who have a poor nutritional status. They remained in the clinic for an average of 19.5 ± 15.7 (median=15.0) days. Accordingly, the length of stay in hospital was significantly extended by about one week on average for malnourished patients.

**Figure 5:**
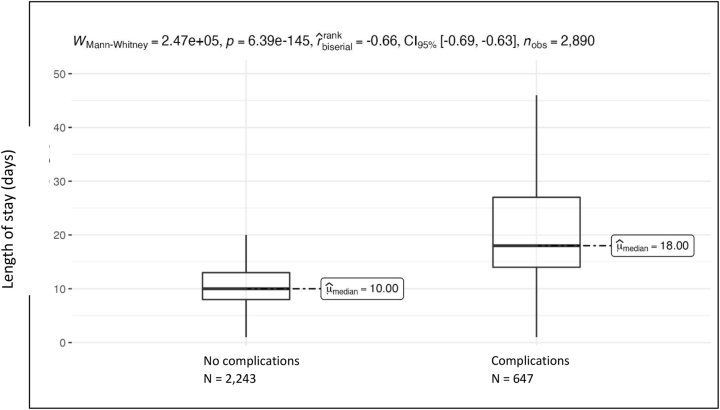
Length of stay as a function of the presence of complications, p<0.001.

**Figure 6:**
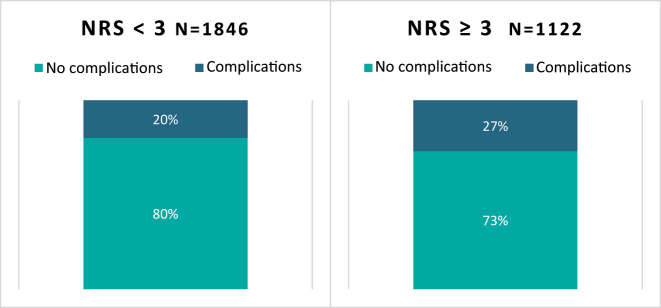
Rate of complications as a function of the determined NRS score, p<0.001, CI _95 %_ [0.06, 1.00].

**Figure 7:**
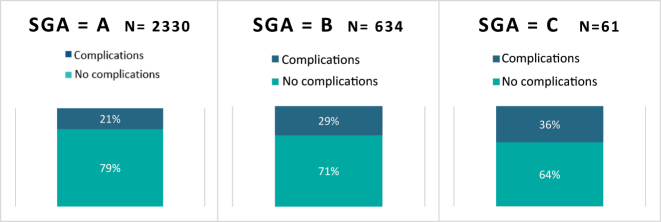
Rate of complications as a function of the determined SGA score, p<0.001, CI _95 %_ [0.06, 1.00].

**Figure 8:**
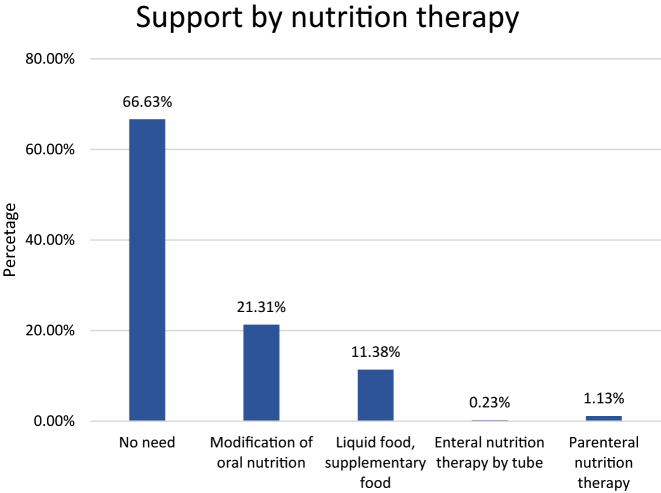
Post-hospitalization interventions with respect to possible nutrition therapy.

### Complications

The complications documented in this study included anastomosis insufficiency (4.6 %, 142), re-operation (3.62 %, 112,), and death of the patients (1.68 %, 52). Further complications were combined under “Other complications” and affected 17.31 % (537) of the patients.

A complication-free course was documented for 76.95 % (2,387).

The average length of stay of patients with complications was 22.9 ± 15.6 (median=18.0) days and was therefore approximately twice as long as the stay of patients who had no complications of any kind (22.9 vs. 11.2) (Figure 5).

Patients with an NRS score of ≥3 had a significantly higher rate of complications compared with the patients who had no risk of malnutrition (27 vs. 20 %) (Figure 6).

Patients who were malnourished according to the SGA screening also experienced complications more frequently (29 % or 36 vs. 21 %). In the patients with an SGA of C (poor nutritional status), one-third developed complications (Figure 7).

### BMI

Most patients (66.2 %) in this study were overweight. Only approximately 1.6 % was underweight. The classification according to the WHO was added [17].

Of the patients who were overweight, 63 % had an NRS score of ≥3.

30 % of the overweight patients had an SGA value of C.

According to the evaluations of this study, malnourished patients remained in hospital longer than patients with a normal weight or overweight patients (median: 16.93 vs. 14.3 or 13.51).

Complications occurred more frequently in underweight patients than in patients with normal weight or overweight patients (30 vs. 24 % or 23 %).

### Support by nutrition therapy during post-hospitalization care

In over 66 % of the patients, no interventions for nutrition therapy were organized in the post-hospitalization phase. The need to modify the oral nutrition was identified in approximately 21 % of patients. The need for liquid food or supplementary food was documented in a further 11 % (Figure 8).

No need for post-hospitalization transfer care was determined in 75.85 %. 17.31 % of the study patients were transferred to their primary care physician or a specialist. Approximately 1.8 % was transferred to an external cooperating partner.

## Discussion

### Prevalence and incidence of colorectal carcinoma

The age-standardized incidence of colorectal carcinomas has declined by 22.4 % (65.3 vs. 50.7 per 100,000) in men and by 25.5 % (42.7 vs. 31.8 per 100,000) in women. Whereas the incidence of carcinomas in the distal colon and rectum has decreased by 34.5 and 26.2 % respectively in men and by 41.0 and 27.9 % respectively in women, the incidence of proximal colon carcinomas in men has remained constant. In women, it has reduced by only 7.0 %. For proximal carcinomas, however, a considerable shift in the incidence to earlier stages has been observed [19].

### BMI

In this study, the BMI was used to subdivide the population into various body-size groups. According to the evaluations of this study, patients defined as malnourished according to BMI remained in hospital longer than normal-weight or overweight patients (median: 16.93 vs. 14.3 or 13.51).

Of the patients who were overweight, 63 % had an NRS score of ≥3. 30 % of the overweight patients had an SGA value of C. It is therefore evident that overweight patients may also be affected by malnutrition.

The evaluations of this study indicated that malnourished patients remained in hospital longer than patients in the normal weight range or overweight patients. Thus, being underweight may be an indicator of the presence of malnutrition.

In addition, complications occurred more frequently in underweight than in normal-weight or overweight patients. According to the results of this study, however, the BMI appears to have less influence on the rate of complications. In contrast, this could be demonstrated with both NRS screening and SGA screening. This again emphasizes the fact that the BMI alone probably has no informative value regarding nutritional status.

### SGA and NRS screenings and BMI

On the basis of the SGA questionnaire, 23 % of the patients had malnutrition (SGA B and SGA C). According to the NRS screening method, the prevalence of malnutrition in the investigated patient group is 38 %. The latter is similar to the study results of Hébuterne et al. [5]. They found the prevalence of malnutrition in colorectal carcinoma to be 39 %. However, evaluation of the BMI was cited as their methodology.

It is obvious that the two screening methods estimate patients quite differently regarding possible malnutrition. Malnutrition is identified by both methods in only 18 %.

In this present analysis, NRS screening identifies a clearly higher prevalence of malnutrition. In other studies, however, the identification rate using the SGA tool is sometimes higher [20, 21].

In the current survey catalog for colorectal cancer centers, the NRS screening tool is clearly recommended for assessing the metabolic risk [2]. In contrast, the comparability seems to be quite different. For example, some studies were able to prove that malnutrition was assessed similarly using both methods [18, 20]. Other studies, such as that of Demirel and Atasoy [22], show a more moderate comparability between NRS and SGA.

It must be added here that a certain amount of briefing or even training should be provided to use this screening tool. This is the only way to ensure uniform implementation and interpretation. In view of the number of participating centers, it is entirely possible for the screening results to vary among the large number of different investigators.

The results of this study show how high the percentage of malnourished oncological patients is in the inpatient sector. The size of the random sample in this study makes it a representative body of data for the German healthcare system. The present survey was designed to collect information about the reality of care and the need for services that provide nutrition medicine. Screening for malnutrition should therefore be firmly anchored in the structures of the colorectal cancer centers. The clinical significance of malnutrition and the benefit of nutritional intervention for patients with cancers have critical relevance. In such cases, malnutrition may occur both as a complication of the underlying disease and as a consequence of certain therapeutic procedures. The negative health consequences of malnutrition and the increased treatment costs resulting from them have been effectively demonstrated [23], [24], [25], [26]. Accordingly, oncology and surgery therapeutic guidelines require systematic and regular screening for malnutrition both at the time of diagnosis and over the further course of the disease [27].

A nutrition assessment usually comprises a nutrition history (qualitative and quantitative food intake), body composition (e.g., using bioelectric impedance analysis), and laboratory parameters. The indication for nutrition therapy is derived from this assessment [28]. Awareness of and regularly reviewing the nutritional status also have implications for cancer therapy, because poor nutritional status may lead to reduced compliance with cancer therapies (chemotherapy, radiotherapy) and may also diminish the response to such therapies [29, 30].

Based on the standardized procedure and the study participation, the study provides a valid overview of the frequency of the risk of developing malnutrition in cancer patients with a colorectal carcinoma. Future investigations should incorporate not only the inpatient sector but also the outpatient sector.

### Complications

The results of this study show that the complications in malnourished patients are significantly greater than in patients without malnutrition. This result is indicated by both NRS screening and SGA screening.

Complications in turn also led to a significantly prolonged length of stay.

Malnourished patients thus demonstrably have a longer length of stay in hospital. This is consequently associated with higher costs and thus has a considerable economic impact.

### Length of stay

Malnourished patients have a significantly longer length of stay: between 3 and 7 days longer, depending on screening method. As early as 2006, Pirlich et al. were able to show similar results in their study. In their study, the length of stay of malnourished patients was 4.6 days longer on average [4].

For patients in the present study who suffered from complications, the length of stay was twice as long compared with patients who had a complication-free course.

For patients who received nutrition therapy preoperatively, the length of stay was 2 days longer on average. In principle, it can be assumed that these patients already had a poorer general condition preoperatively, leading in turn to a prolonged stay. The longest length of stay was found for patients who received parenteral nutrition preoperatively. However, it can be assumed that the difference in lengths of stay would be even greater without preoperative procedures. The patient would undergo surgery with an even poorer initial situation, which in turn could result in an even poorer outcome.

### Interventions providing nutrition therapy and transfer

Despite the increased prevalence of malnutrition, 66.6 % of the patients was still being discharged to the post-hospitalization setting with no description of a need for nutrition therapy. In this context, it would be interesting for the future to analyze the degree to which additional post-hospitalization interventions affect the further course of malnourished patients.

There was no need for post-hospitalization transition care identified for over 75 % of the included patients. 17.3 % of the study patients were transferred to their primary care physician or a specialist. Only 1.8 % was transferred to external cooperating partners, such as homecare providers. In particular, interdisciplinary support as well as cooperation in the outpatient sector may have a decisive influence on subsequent convalescence.

Procedures providing nutrition medicine and their implementation are explicitly described in the guidelines. In addition, advice is included about training of patients or their relatives, for example, by homecare providers [31].

The available screening methods can be carried out quickly and without great extra expense and can counteract prolonged hospitalizations and the resultant higher costs.

However, prior training is recommended to ensure that the methods are interpreted and implemented uniformly.

## Summary

In this study, the prevalence of malnutrition was 38 % when using the NRS-2002 score (≥3 points) and 23 % when using the SGA. According to the results of NRS screening, therefore, at least one-third of the patients in colorectal cancer centers were suffering preoperatively from malnutrition.

More patients were classified as malnourished by the NRS score. This may be explained by the fact that this score identifies the risk of malnutrition. In contrast, the SGA score divides the patients into three groups (well, moderately, and poorly nourished).

According to this study, malnourished patients have a significantly higher rate of complications. Patients with complications have a significantly longer length of stay than patients without complications.

The data generated by this study clearly show how high the rate of malnourished patients is in colorectal cancer centers. A higher postoperative rate of complications and longer lengths of stay are the consequence. This justifies the need to conduct systematic screening for malnutrition and to provide nutrition therapy for patients at risk of malnutrition.

Accordingly, nutrition management supported by specialists is indispensable. Even within the context of prehabilitation, nutrition should be given greater importance and thus support the recovery of the patients.

Systematic screening for malnutrition is not yet adequately implemented nor is nutritional advice that complies with the guidelines. Such screening needs to be more widely accepted and established in routine clinical practice. This leads to an urgent call for action to close existing loopholes in providing care. Systematic screening for malnutrition and, if necessary, early nutritional intervention should be an obligatory component of the support provided for patients with cancer. This can demonstrably reduce possible complications, leading in turn to a shorter length of stay and thereby to cost reductions.

In accordance with the requirements for colorectal cancer centers, the importance of early detection of malnutrition in patients with colorectal cancer is confirmed with this study.

Thanks to this study we were also able—according to statements from some centers—to further expand the issue of clinics’ internal nutrition management. Due to regular screening, medical staff became more aware of possible malnutrition and possible consequences and interventions were discussed at the interdisciplinary level in the centers.
